# Mixture Effects Investigation of a Thyroid Hormone System-Specific Interaction with Azole Fungicides

**DOI:** 10.3390/toxics14090749

**Published:** 2026-08-25

**Authors:** Asya Kadic, Anne Elisabeth Reetz, Benjamin Christian Fischer, Boubacar Sidiki Sylla, Katreece Feiertag, Vera Ritz, Tanja Heise, Philip Marx-Stoelting, Tewes Tralau, Marize de Lourdes Marzo Solano

**Affiliations:** 1 German Federal Institute for Risk Assessment, Department of Pesticides Safety, 10589 Berlin, Germany; asya.kadic@bfr.bund.de (A.K.); vera.ritz@bfr.bund.de (V.R.); tanja.heise@bfr.bund.de (T.H.);; 2 Institute of Veterinary Pathology (WE12), Freie University Berlin, 14163 Berlin, Germany

**Keywords:** mixture effects, thyroid hormone pathways, adverse outcome, pesticides, azole fungicides

## Abstract

This study investigated the thyroid effects of azole fungicide mixtures in male rats, addressing the gap in regulatory assessments that typically focus on single chemicals rather than combined exposures. Using a 28-day rat model, binary and ternary mixtures of azole fungicides were tested at high doses, with thyroid hormone levels, liver enzyme activity, and thyroid tissue changes as the key endpoints. The mixtures caused elevated TSH, reduced T4, increased UGT enzyme activity, and thyroid tissue alterations, including follicular cell hypertrophy and hyperplasia. Interestingly, the mechanism observed with single-compound exposures (hepatic metabolism driving secondary TSH elevation) appeared less prominent in the mixture groups. Whether these responses reflect additive, less-than-additive, or more-than-additive interactions could not be determined without formal dose–response modeling. As the tested dose (NOAELx10) is substantially higher than the human realistic exposure levels permitted under the current regulation, direct extrapolation of the present findings to realistic human exposure conditions is limited. However, the current regulatory evaluation with respect to the effect dose was confirmed. While existing approaches appear adequate, a deeper understanding of thyroid-specific endpoints is needed to ensure that mixture risk assessments remain robust and appropriately protective.

## 1. Introduction

Risk assessment of plant protection products (PPPs) requires consideration of all substance interactions with potential toxicological significance, as stipulated by Regulation (EU) No. 1107/2009. Despite the comprehensive nature of existing legislation, it predominantly emphasizes the effects of individual substances. Although this approach has served well so far, it is not necessarily representative of real-life exposure scenarios, which usually comprise intended and unintended mixtures [1]. Although current health data do not provide evidence for immediate concern, and claims of highly synergistic interactions usually have little toxicological merit, the possibility of mixture effects should not be readily dismissed [2]. Such effects have been observed, particularly for chemicals with similar mechanisms of action [3,4]. The European Food Safety Authority (EFSA) and other regulatory bodies have sought to develop strategies to address mixture toxicity assessment [5]. Traditionally, these assessments have used a toxicodynamic approach based on the concept of dose addition (DA), where the combined effects of chemicals are presumed to be additive when they share a common target organ or mode of action [6]. Consequently, these groups can be categorized into cumulative assessment groups (CAGs) [7].

Pesticides, along with pharmaceutical drugs, possess some of the most extensive regulatory datasets, making them ideal for further investigation into the molecular or kinetic aspects of toxicology, as well as conceptual case studies. Several pesticide mixtures, including azoles [8], have been studied in the context of thyroid disruption [9,10]. The thyroid gland is part of the endocrine system that produces thyroid hormones (THs). These hormones and the overall thyroid function are crucial for the neurological and physiological development of the fetus and play an important role in metabolic rate, stimulation of new protein synthesis and turnover, cardiovascular, neurological, and skeletal systems, immunity, and communication with other hormones [11,12,13]. Circulating thyroid levels are tightly regulated by thyroid-stimulating hormone (TSH), which operates in a negative feedback loop with TH [14]. Alterations in the metabolism of thyroid hormones can significantly impact the levels of active TH available, potentially leading to adverse health effects in the early as well as later stages of life. This makes the hypothalamic-pituitary-thyroid (HPT) axis a sensitive target and is of particular interest in the context of the potential thyroid disruption (TD) properties of pesticides.

In 2019, EFSA published a cumulative risk assessment (CRA) report in which they established CAGs of pesticide active substances (AS) sharing a common mode of action (MoA) or target organs for two thyroid toxicity endpoints: hypothyroidism and parafollicular cell (C-cell) hypertrophy, hyperplasia, and neoplasia. Of the 400 AS evaluated, 128 were included in the hypothyroidism CAG, including cyproconazole, among other azole fungicides [15]. The 2024 EFSA update confirmed the two thyroid-related CAGs that were previously defined while refining the list of indicators. For hypothyroidism, histopathological changes are now the defining indicators, with serum hormone and thyroid weight changes serving as supporting evidence rather than primary indicators. Additionally, the criteria for CAG inclusion were reviewed and refined [16]. Azole fungicides have been used for more than 40 years and are among the most commonly used fungicides. These fungicides function by inhibiting the CYP51 enzyme that is involved in cell membrane permeability and fluidity [17,18]. Consequently, EFSA has selected them as model compounds for constructing a CAG for chronic exposure [19].

**Figure 1:**
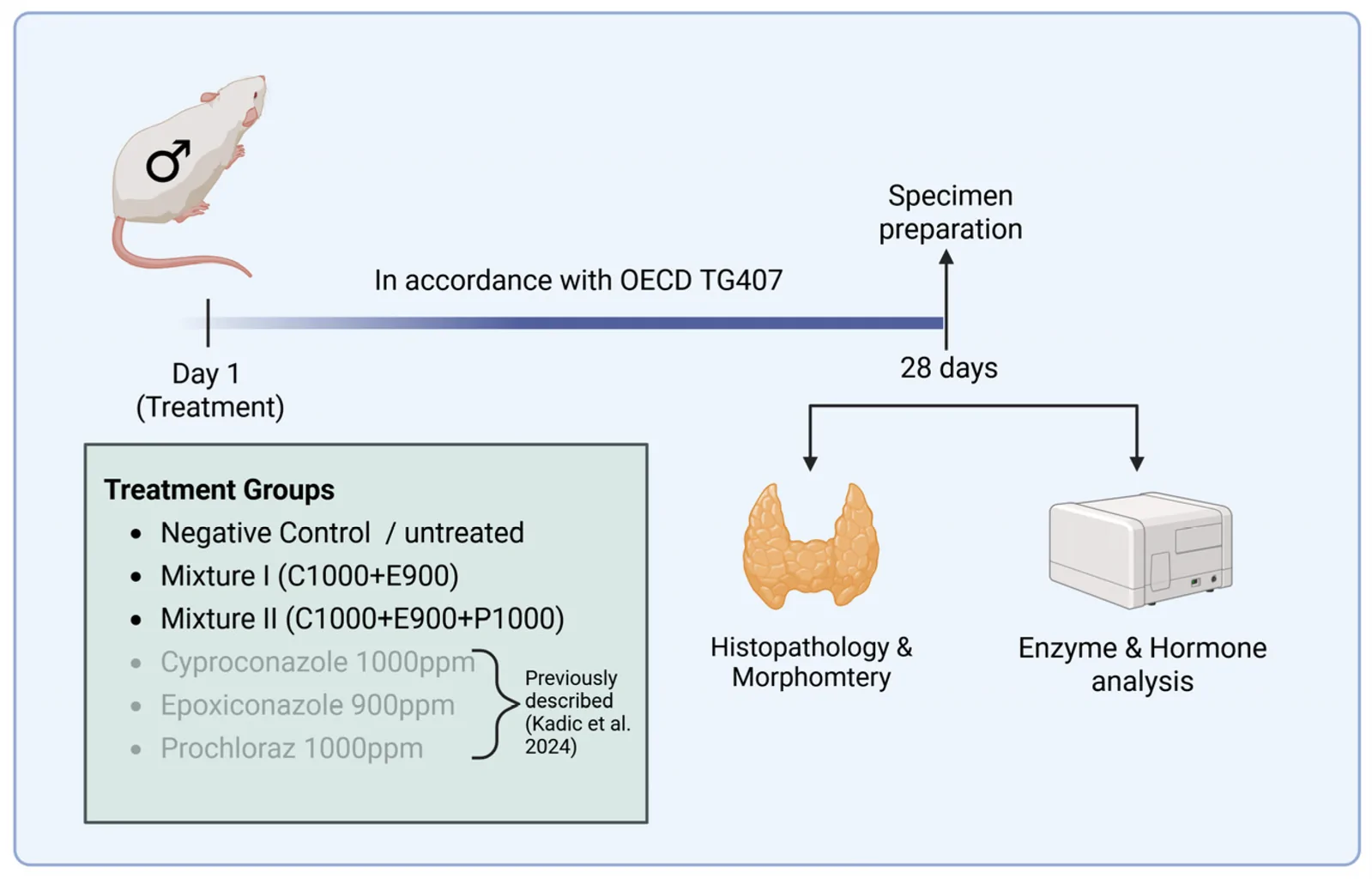
Experimental set-up and design [23].

The toxic effects of three azole fungicides—cyproconazole (C), epoxiconazole (E), and prochloraz (P)—have been previously investigated in the liver and other organs, both individually [20] and in mixtures [21,22]. The effects of individual azole fungicides on the thyroid were also explored in a 28-day study, complemented by in vitro, in chemico and ex vivo assays to examine their mode of action on thyroid function and gland [23]. In the present follow-up study, we examined the binary and ternary mixture effects of C, E, and P at high doses over 28 days, with a comprehensive evaluation of thyroid histology, morphometry, serum TH and TSH levels, and hepatic UGT enzyme induction. Figure 1 shows a summary of the experimental design.

It should be noted that the experimental work was performed at a time when the investigated substances were still approved in the EU. While these pesticides C, E and P are no longer approved, the data remain relevant for mechanistic understanding of thyroid-related effects of azole fungicides. This case study, therefore, aims to provide insight into mixture effects on the HPT axis and improve understanding of azole toxicity.

## 2. Materials and Methods

### 2.1. Test Substances

Hepatotoxic fungicide cyproconazole (C) (CAS no. 94361-06-5, purity 96.8%) was obtained from Syngenta (Basel, Switzerland). Epoxiconazole (E) (CAS no.133855-98-8, purity 97.0%) and prochloraz (P) (CAS no. 67747-09-5, purity 98.0%) were supplied by BASF (Ludwigshafen, Germany). The test substances from manufacturers were mixed into rodent diet R/M-H V155 by Ssniff (Soest, Germany), and their concentration was checked by SGS Fresenius (Berlin, Germany) using method ASU L. 00.00-115 LC under GLP conditions. The control diet was checked for the absence of pesticides, especially triazole fungicides. T3 and T4 were obtained from Sigma-Aldrich (Hamburg, Germany). Chemicals for in vitro tests were obtained from commercial sources in sufficient quality.

### 2.2. Animal Experiments

A 28-day feeding study with male Wistar rats examined fungicide mixtures of cyproconazole, epoxiconazole, and prochloraz. Animals were 9 weeks old at treatment onset, group-housed (5/cage) under standard facility conditions (20 ± 2 °C, 50–60% humidity, 12 h dark/light cycle), and monitored daily for clinical signs, with body weight recorded weekly and food consumption measured daily per cage. The principles of the experiment and parameters analyzed were in accordance with OECD TG407, with the exception of the experiment was conducted with male animals only, since males were shown to be slightly more sensitive in previous studies used within the approval procedure for the respective fungicides. Therefore, the study design was kept the same as previous studies, consistent in this series [20,21]. In short, ten animals were assigned to each mixture group (I and II), and 15 animals were assigned to the negative control group. Dose selection was based on NOAELs from feeding studies up to 10 × NOAEL, in which hepatic effects (increased liver weight and hepatocellular hypertrophy) were observed predominantly at the highest dose level, while lower doses (down to NOAEL/100) showed no adverse effects on the body weight, food consumption, or clinical chemistry. The highest dose was therefore chosen to maximize sensitivity for detecting potential thyroid effects, consistent with the results of Kadic et al. [23]. In mixture groups, each component was administered at the same concentration as in single-compound exposures, with the total mixture dose representing the sum. Mixture group I contained cyproconazole (1000 ppm, ca. 74 mg/kg bw/day) and epoxiconazole (900 ppm, ca. 62 mg/kg bw/day), while mixture group II included prochloraz (1000 ppm, ca. 68 mg/kg bw/day). Data from the highest-dose single-compound groups were included for comparison. Dietary concentrations and mean intakes are detailed in the Supplementary Information.

At the end of the treatment period, the animals were deeply anesthetized with Sevofluran (Abbott, Wiesbaden, Germany), cardial blood was sampled by use of a 21G Sangocan blood sampling canule (Kabe Labortechnik, Nümbrecht-Elsenroth, Germany) and animals were finally sacrificed in 95% CO_2_ and 5% O_2_. All experimental procedures were performed in accordance with the relevant ARRIVE guidelines and regulations.

### 2.3. Microsome Isolation

Approximately 1 g of unfrozen rat liver was homogenized in 250 mM sucrose solution containing 1 mM EDTA using a Potter-Elvehjem tissue grinder (400 rpm) (Sigma Aldrich, Taufkirchen, Germany). To keep the microsomal enzymes intact, the liver was cooled during homogenization. The homogenate was centrifuged for 10 min at 2500 rpm (420× *g*) at 4 °C. In case a fatty layer formed, it was removed carefully using a pipette. The microsomes containing the supernatant were decanted into an ultracentrifuge tube, and the pellet was discarded. The supernatant was centrifuged for 60 min at 38,000 rpm (97,000× *g*) at 4 °C in an ultra-centrifuge. The supernatant was discarded, and the pellet was washed by resuspending it in 150 mM KCl solution and again ultra-centrifuged for 60 min at 38,000 rpm (97,000× *g*) at 4 °C. Finally, the pellet was resuspended in 250 mM sucrose solution (without EDTA), and aliquots were taken for protein content determination or stored at −80 °C for later use. Protein concentrations were determined using the bicinchoninic acid (BCA) assay, according to the manufacturer’s protocol.

### 2.4. UGT Activity Assay

UGT activity analysis was conducted using the UGT Activity Assay Ligand Screening Kit (BioVision- Abcam, Milpitas, CA, USA, K692-100, Lot 6F15K06920) following the manufacturer’s protocol. In summary, reaction mixtures were added to 2 µg microsomes per treatment group per animal (*n* = 5) in duplicates and were incubated for 5 min at 37 °C protected from light. Finally, 5X UDPGA substrate was added to each sample well, and fluorescence (Ex/Em = 415/502 nm) was immediately measured in kinetic mode for 40 min at 37 °C using a Tecan Plate Reader (Tecan Group Ltd., Männedorf, Switzerland) and software (Tecan i-control, Infinite 200Pro, Version 1.5). One unit of UGT activity represents the amount of enzyme that glucuronidates 1 µmol of fluorescent substrate per minute (yielding a nonfluorescent conjugate) at 37 °C and pH 7.5.

### 2.5. Serum TH and TSH Measurements

Total TH levels (tT3, tT4) and TSH in rats after 28 days of treatment with mixture groups I (cyproconazole, and epoxiconazole) and II (cyproconazole, epoxiconazole, and prochloraz) were analyzed by MagPix multiplex assays (MILLIPLEX^®^ Rat Thyroid Magnetic Bead Panel—Endocrine Multiplex, Cat # RTHYMAG-30K, Kit Lot # 3278791). Multiplex measurements from 25 µL serum per replicate (*n* = 5) in duplicates by fluorescence were conducted using a Bio-Plex MAGPIX Multiplex Reader (Luminex Corporation, Sioux Falls, SD, USA), according to the manufacturer’s instructions.

### 2.6. Histopathology and Microscopy

One thyroid lobe per animal was bisected transversely at parathyroid gland level, creating anterior and posterior sections. Both sections were blocked using Tissue-Tek O.C.T. and frozen at −80 °C. From each section, 2–3 serial 7 µm slices were prepared (6–9 sections per animal) using a Thermo Scientific Cryostat HM550. Slides were fixed with 10% formalin and prepared using the standard H&E staining protocol. H&E-stained thyroid slices were evaluated under Zeiss Axio Observer D1 microscope with halogen brightfield illumination at 50–200× magnification. Images were captured digitally and processed using Zeiss Zen Software 3.4. Histopathological evaluation examined follicular hypertrophy, hyperplasia and dilation using a semi-quantitative scoring system in line with toxicological pathology practice [24]. Scoring was performed blindly by three observers using the Standardized System of Nomenclature and Diagnosis Criteria [25], referenced to corresponding negative (not treated).

### 2.7. Thyroid Morphometry Analysis

**Figure 2:**
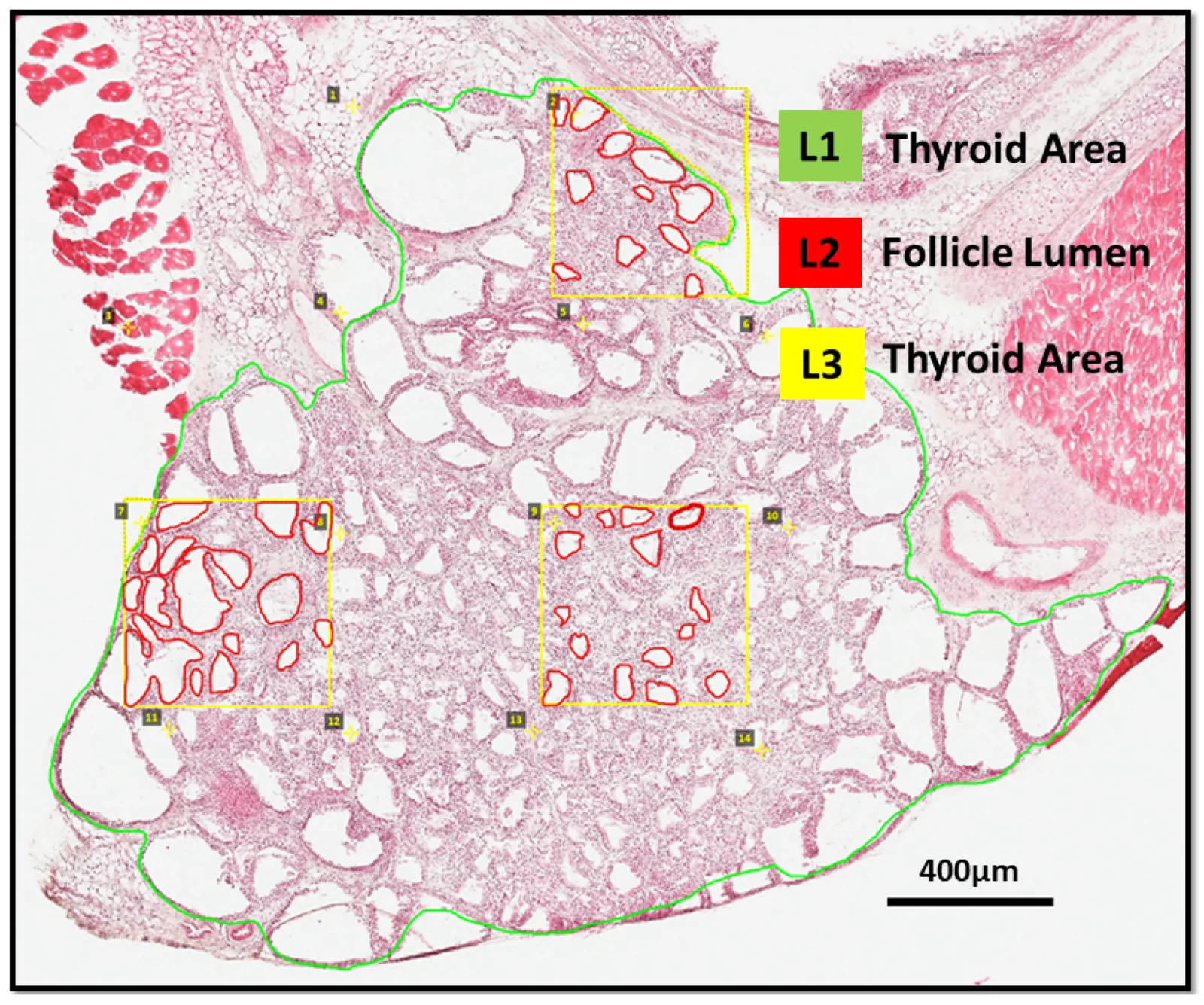
Thyroid morphometry analysis using ImageScope software.

Thyroid sections from 4 to 6 animals per group were used, and from each animal, two representative central sections of one thyroid lobe were selected for digital evaluation, as these regions represented the whole lobe. Slides were digitized using the Aperio CS2 Scanner (Leica Biosystems Imaging Inc., Vista, CA, USA) at 400× magnification (0.25 micrometers/pixel) and evaluated using Aperio ImageScope x64 and QuPath v0.4.3 software. Annotated thyroid regions were quantified for total tissue area, high-power field (HPF) area, follicle number, follicular lumen area, and epithelial height. Non-thyroid tissue was excluded from analysis. Measurements were performed in a blinded fashion by two independent observers and averaged per animal before group comparisons. A glossary of morphometric parameters and calculations is provided in Table 1, and the corresponding areas examined are in Figure 2. Further morphometric information is provided in the Supplementary Information.

### 2.8. Statistical Analysis

For parametric and nonparametric standard tests, the SigmaPlot for Windows software (Version 11.0, Systat Software Inc., San Jose, CA, USA, 2008) and statistical tests suggested by the software were used. For hormone, UGT enzyme and morphometry analysis, an ordinary one-way ANOVA was applied first. Then, the normality of the residuals (Shapiro–Wilk test) and the homogeneity of variance (Bartlett’s and Brown-Forsythe tests) were assessed at *p* ≤ 0.05. If all tests pointed towards both assumptions being fulfilled, the result of the ordinary one-way ANOVA was considered, and if significant at *p* ≤ 0.05, followed by a Dunnett’s post hoc to compare treatment groups against the negative control. In cases where assumptions were not met, the non-parametric Kruskal–Wallis test was used, and if significant at *p* ≤ 0.05, followed by a Dunn’s post hoc test. The software applies a built-in correction for multiple testing to reduce Type I error. For histopathological data, the negative control and treatment groups were compared using Fisher’s exact test. Differences were considered statistically significant for * (*p* ≤ 0.05) or ** (*p* ≤ 0.01).

## 3. Results

### 3.1. Thyroid Hormone Metabolism

**Figure 3:**
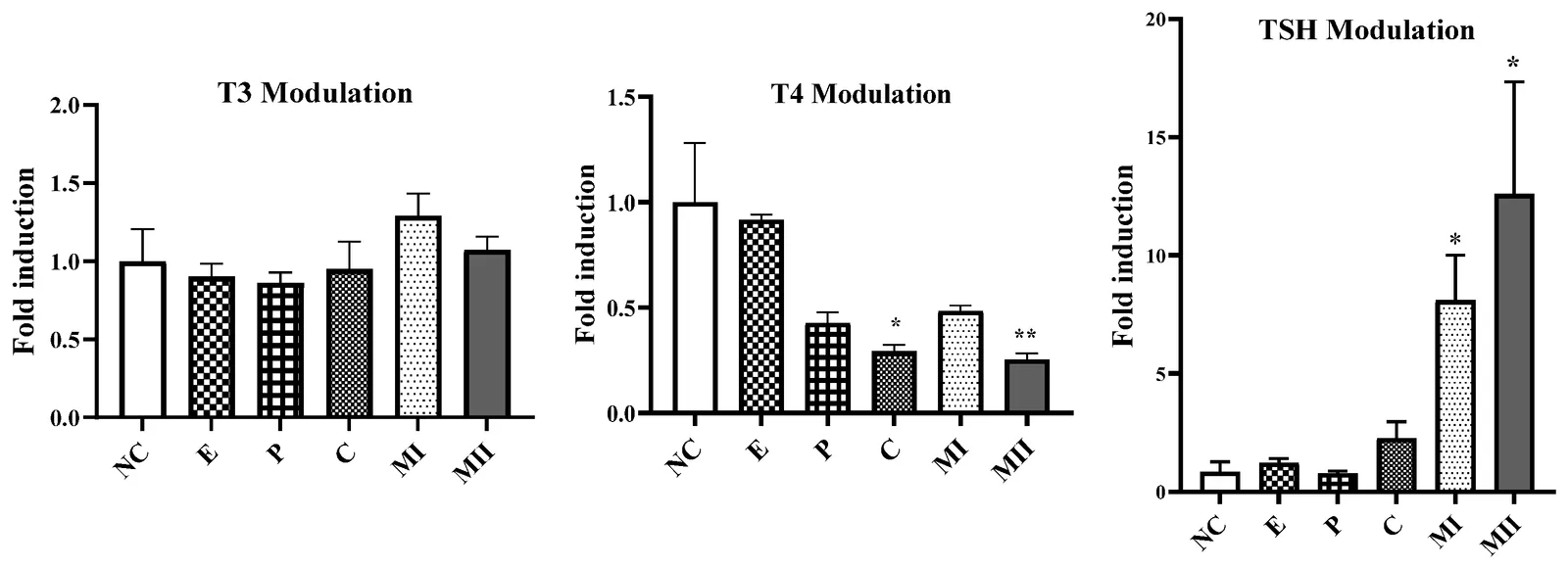
Modulation of thyroid hormone levels T3, T4, and TSH in rats, measured in serum taken after 28 days of treatment (*n* = 5). Values are given as fold modulation relative to the controls ± SEM (one-way ANOVA with * *p* ≤ 0.05 or ** *p* ≤ 0.01). Abbreviations as follows: NC = negative control, E = epoxiconazole, P = prochloraz, C = cyproconazole, MI = mixture I/binary mixture (C + E), MII = mixture II/ternary mixture (C + E + P). Individual substances C, E, and P were derived from the previous study by Kadic et al. [23] for comparison.

Total TH levels and TSH were analyzed using MagPix multiplex assays to determine whether azoles affected serum hormone levels (Figure 3). For comparison purposes, the individual chemical exposures for C, E, and P were included along with mixtures. A decrease in serum T4 levels was observed in all treatment groups, except for the epoxiconazole group. It should be noted that while the results were statistically significant for treatment with the ternary mixture (MII) and for cyproconazole, they were not significant for prochloraz or the binary mixture (MI) of epoxiconazole and cyproconazole. The TSH levels increased significantly in both mixtures, with the highest levels of production in the ternary mixture group compared to the single-substance treatments, where no increase was observed. No changes were observed in T3 levels.

### 3.2. UGT Enzyme Activity

**Figure 4:**
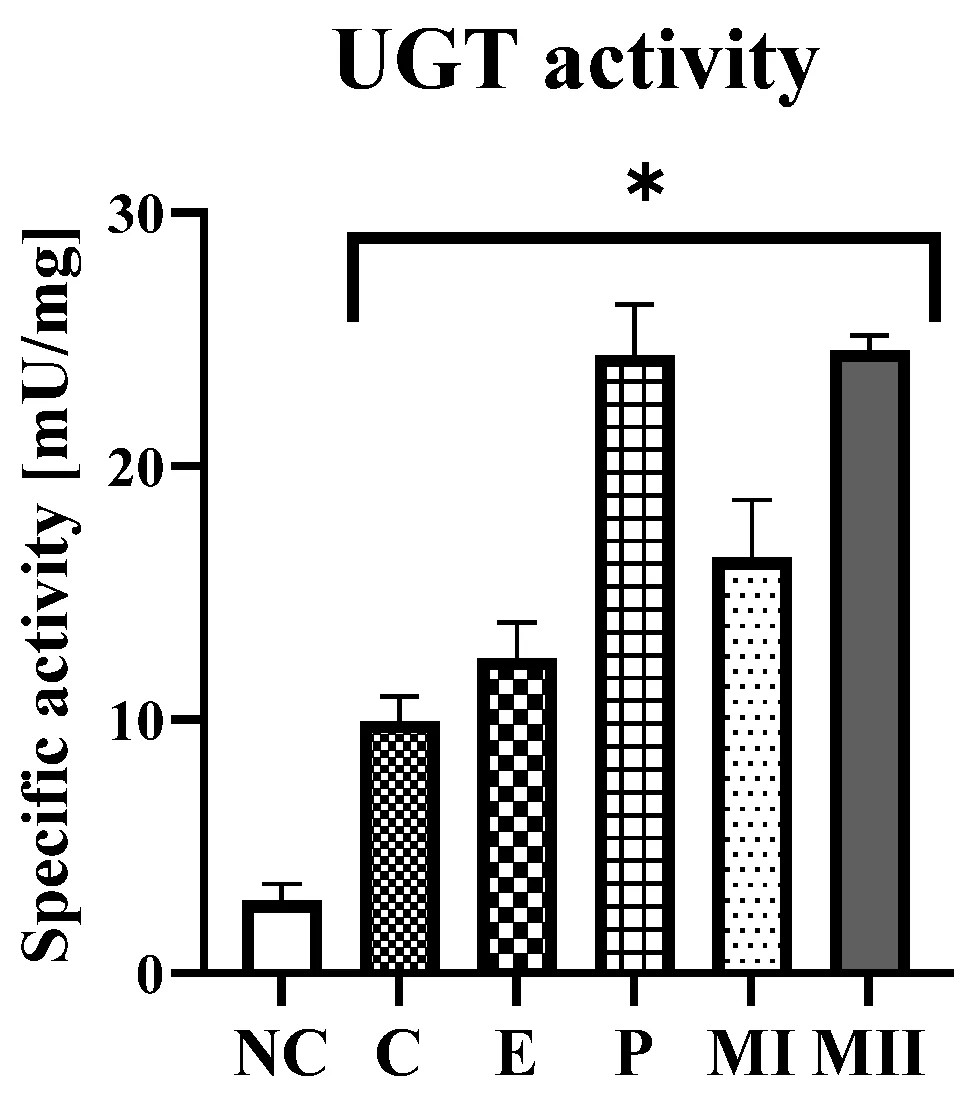
UGT activity across binary and ternary mixture treatment groups (data are expressed as mean ± SEM) (*n* = 5). (One-Way-ANOVA * *p* ≤ 0.05). One unit of UGT activity represents the amount of enzyme that glucuronidates 1 µ mole of fluorescent substrate per minute (yielding a non-fluorescent conjugate). Abbreviations are as follows: NC = negative control, C = cyproconazole, E = epoxiconazole, P = prochloraz, MI = mixture I/binary mixture, MII = mixture II/ternary mixture. Individual substances C, E, and P were derived from the previous study by Kadic et al. [23] or comparison.

To investigate the effect of hepatic metabolism of TH by UGTs, an enzyme activity assay was performed, focusing on a combination of isozymes, including UGT 1A1, 1A3, 1A6, 1A9, and 2B7 (Figure 4). UGT activity was significantly induced across all treatment groups, as shown previously by Kadic et al. 2024 [23]. The same pattern holds for mixtures in this study. Notably, UGT activity was most prominent in P and MII, which showed similar values, suggesting that prochloraz may be a major contributor to the elevated activity observed in the ternary mixture group. MI also showed higher activity than either C or E individually, indicating that a potential mixture interaction may be taking place. However, these comparisons were not directly statistically tested, and further analysis would be needed to confirm these relationships. Overall, the induction observed across all treatment groups aligned with the reduction in T4 and increase in TSH mixtures, although the relationship was less clear for individual chemicals.

### 3.3. Thyroid Histopathology and Morphometry

To assess histopathological changes in the thyroid gland, follicular hypertrophy, hyperplasia, and dilation of the thyroid glands were recorded and graded using a semi-quantitative scoring system to capture the incidence and severity [24]. Table 2 provides an overview of the histopathological observations in the thyroid gland, with data represented as the number of animals with findings/total number examined (percentage). Follicular hypertrophy and hyperplasia were observed in both mixture groups but not in the control group. The incidence of hypertrophy and hyperplasia was the same in both mixture groups, with no prominent difference in incidence in either group. Follicular dilation was present in the negative control group as well as in both mixture groups. Finally, only two of the five animals in the negative control group had no visible lesions, and visible lesions were present in all animals in both mixture groups.

**Figure 5:**
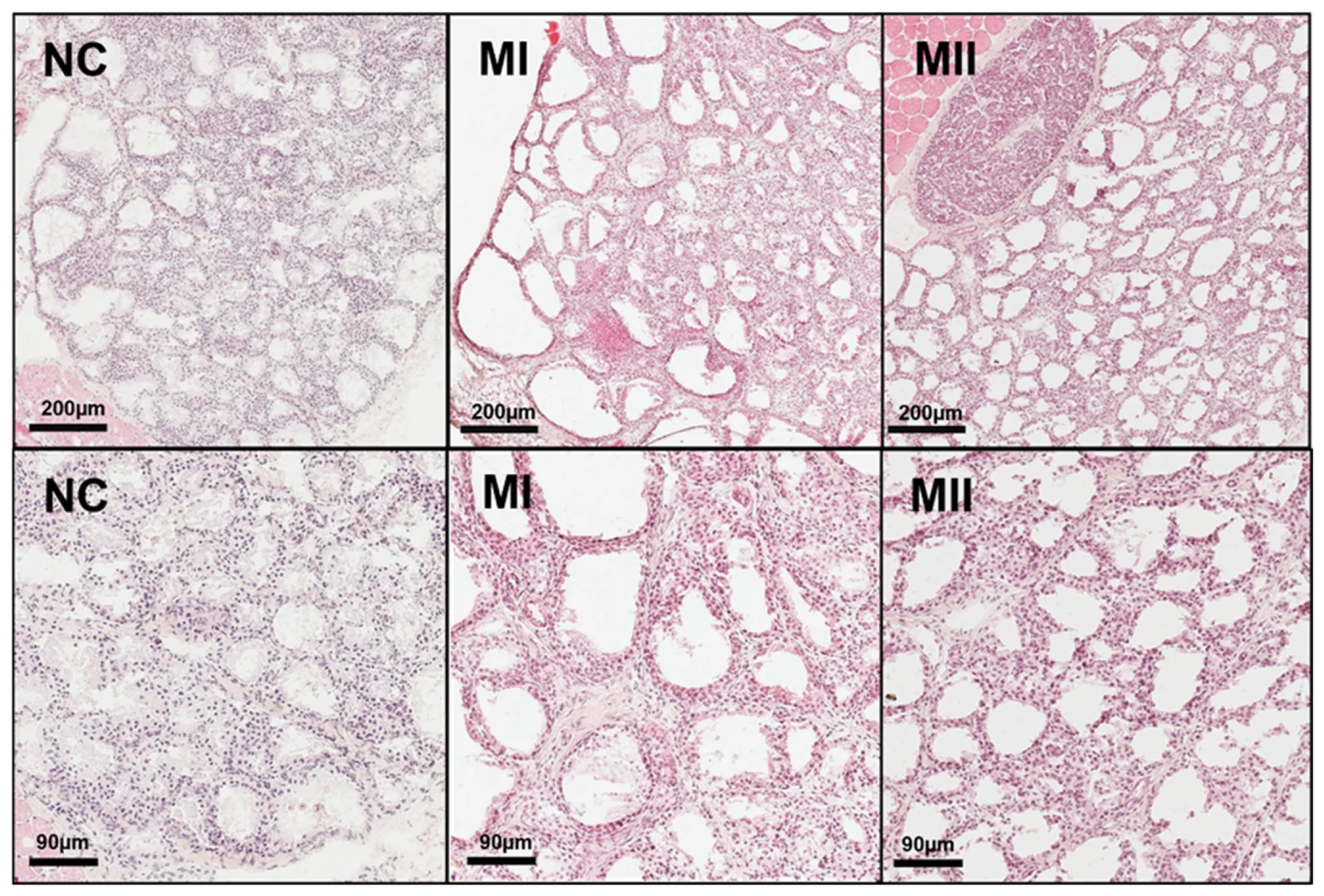
Thyroid follicular hyperplasia and hypertrophy across mixture treatment groups compared to the negative control. Images are representative of a tissue sample from one of the *n* = 5 animals examined. First row at 10.8× magnification (scale bar at 200 µm) in the following order: NC—negative control, MI—mixture I group, MII—mixture II group. Second row at 23.2× (scale bar at 90 µm) in the following order: NC—negative control, MI—mixture I group, MII—mixture II group.

As shown in Figure 5, follicular cell hypertrophy was evident in both mixture groups. Focal follicular hyperplasia was observed more than once and was well-defined (Table 2). There was no difference in the signs of follicular hypertrophy and hyperplasia between the azole treatments (all animals). The follicles in the mixture groups were mostly hypertrophic and had a reduced lumen at the center of the gland. The follicle sizes decreased in the central area and were mostly surrounded by dilated follicles at the periphery of the area. The borders were rounded and macroscopically shaped by dilated follicles. An increase in cell height and disappearance of the lumen in some follicles were also observed, although not as often as in the other treatment groups (i.e., mostly the lumen was preserved). These hypertrophic follicles without a lumen were more evident and larger than those in the cyproconazole treatment group.

The severity of the observed histopathological alterations was graded based on a point system (Table 3). Data are presented as the number of animals examined per group, with the incidence of follicular hypertrophy, hyperplasia, and dilation indicated by x-marks (one x per animal). Severity was graded using a semi-quantitative scoring system adopted by Alarcan et al. 2020 [26] (color-coded blue x: slight/1 point; hyperplasia: one to two follicles affected; hypertrophy: not all follicles or only part of the follicles affected; red x: moderate = 2 points; hyperplasia: more than one follicle affected (multifocal); hypertrophy: every follicle or almost all follicles affected). Consistent with the incidence rates and histopathology figures (Figure 5), both mixture groups showed severe histopathological changes, particularly follicular hypertrophy, which received the highest severity score (10 points) in both groups. Hyperplasia also received higher scores (9 points for both mixture groups) than the negative control group, indicating that not all follicles were affected but still showed multifocal hyperplasia. Compared with the individual azole treatment, the overall hyperplasia and hypertrophy severity scores were slightly lower than those in the mixture groups (individual data shown at Kadic et al [23]). Finally, spontaneous slight follicular dilation was observed in the negative control group (Table 3) and in both mixture groups, but with a much higher incidence and larger size.

**Figure 6:**
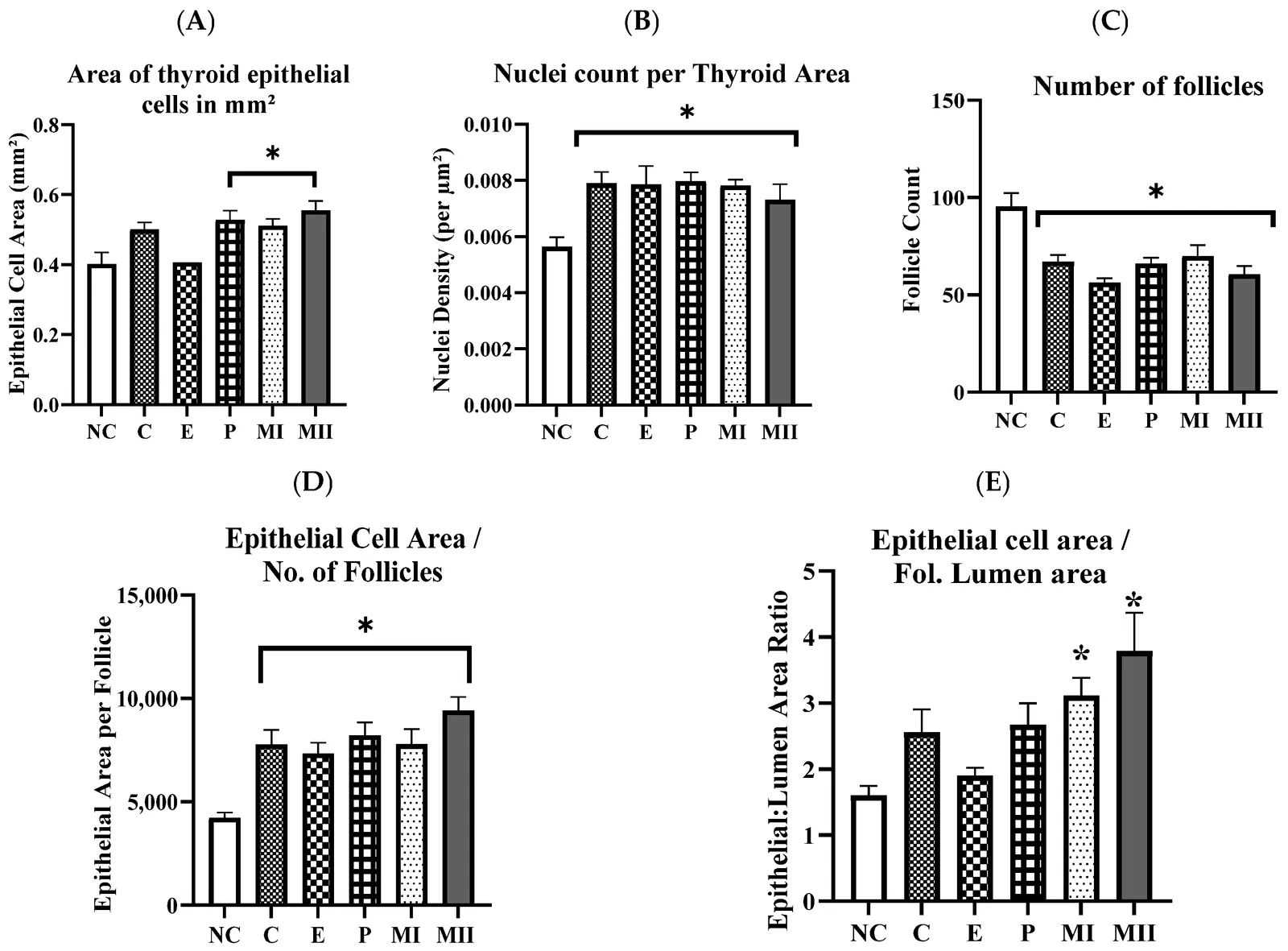
(**A**–**E**) Thyroid morphometric analysis: (**A**)—epithelial cell area (mm^2^), (**B**)—nuclei count per thyroid area (µm^2^), (**C**)—number of follicles, (**D**)—epithelial cell area per follicle, (**E**)—epithelial cell area/follicle lumen area ratio. Values are given as mean + SEM, relative to negative control; * (*p* ≤ 0.05).

Morphometrics measurements were done for both the individual and mixture treatment groups for the first time to quantify the histopathological effects observed in this study. Epithelial cell area differed among the treatment groups, with the highest value in the MII group (Figure 6A). However, the average cell area increased for most substances compared to the negative control. This could indicate hypertrophic or hyperplastic effects on the cells due to the increased area covered by epithelial cells. The smaller pronounced or insignificant results may be due to the temporal aspects of substance exposure. The average nuclear count per unit area was higher for most substances than that for the negative control (Figure 6B). This can be explained by an increase in cell proliferation (hyperplasia), indicating that the first indicators of hyperplasia can be observed after 28 days of treatment. Interestingly, the increase in cell numbers appeared to be similar among the treatment groups. These results indicate that the underlying pathway for hyperplasia in the thyroid differs from that for hypertrophy and that both negative outcomes must be analyzed individually and compared. The number of follicles decreased in most treated groups compared with control (Figure 6C), suggesting potential effects on follicle development or maintenance, but can be interpreted as an increase in cell size and number, hypertrophic or hyperplastic effects that affect the follicle lumen, and a negative change in the number of follicles. Epithelial cell area per follicle (Figure 6D) increased in each treatment group, indicating a lower number of follicles, with the highest values in the MII group. A low value of this ratio represents a high number of follicles per area, indicating a decreased number of follicles in all the treatment groups. The lowest number of follicles was observed in the MII group. Finally, the epithelial cell area per follicular lumen area, also known as the “thyroid activation index”, showed a diverse distribution, with lower values in the negative control and individual pesticides and peaks in the MI and MII groups (Figure 6E). The thyroid activation index increases as the thyroid activity increases. This change reflects an increase in epithelial volume and a concurrent decrease in colloid volume.

## 4. Discussion

This study investigated the combined effects of cyproconazole (C), epoxiconazole (E), and prochloraz (P) on the HPT axis. At high-dose exposures (NOAELx10), binary (C + E) and ternary (C + E + P) mixtures were assessed through histopathological, morphometric, enzymatic, and hormonal analyses to explore the mechanisms and mixture effects. Our findings are also supported by those of Noyes et al. [27] and others, in that thyroid disruption from azole mixtures results as a secondary mechanism of hepatic enzyme induction via CAR and PXR [28,29,30,31]. With respect to the rather high doses, it can be assumed that due to the strict reference levels set for the individual compounds and as exposure via food is several orders of magnitude lower than what is used in regulatory animal testing, no risk is associated with current exposure levels.

With regard to enzyme and hormone analyses, our findings follow a similar pattern of enhanced hepatic catabolism via UGT enzymes, combined with affected TH levels, echoing previous results for single-substance exposures [20,22,23]. We observed UGT enzyme induction across all treatment groups, although it was seemingly less than additive for both mixtures. In contrast, we observed an overall reduction in T4 across all treatment groups (except for E) and an increase in TSH modulation only in the mixture groups. The lack of changes in T3 (as opposed to T4) could be explained by the differences in TH-regulating MIEs. A review by Crofton suggested that the inducers of T4 glucuronidation via hepatic UGT induction tend to have a greater effect on T4 and a lesser effect on T3 and TSH [30,32,33]. Additionally, TH levels are tightly regulated by deiodinase enzymes that control cellular concentrations, where T4 is converted to T3 by deiodinase 1 or 2 (DIO1 and DIO2), which could potentially explain the decrease in T4 as a compensatory mechanism [34]. However, this is unlikely, as our previous results showed no indication of other MIEs being involved [23]. The lack of T3 change also contradicts a study in which T3 glucuronidation was postulated to mediate TSH in rats, although the mechanism was unclear and was postulated to be related to other MIEs [30]. Overall, the most likely explanation for these results is the increased hepatic metabolism of T4 due to the induced UGT activity, followed by a subsequent increase in TSH. Correspondingly, it is important to consider that hormonal changes are subject to such feedback mechanisms. As only a single time point was analyzed (i.e., animals were sacrificed after 28 days), this would not cover all changes expected within a continuous process but provide a snapshot in time only. Given the semi-quantitative nature of histopathological analysis, which is prone to interpretation variability due to subjective scoring [35], we implemented a quantitative morphometric evaluation system to enhance the evaluation. In particular, the “thyroid activation index”, represented as the ratio of epithelial volume to colloid volume and reflecting changes in thyroid function due to TSH alterations, tends to increase when the thyroid gland becomes more active [36]. Our morphometric analysis was in line with the findings of TSH stimulation and subsequent histopathological changes by showing an increase in epithelial cell volume and nuclei per area, indicating thyroid stimulation, which was shown in cases of TDCs in single-substance exposure and/or mixtures. Overall, a standardized morphometric protocol is essential to address the limitations of descriptive pathology in assessing thyroid alterations due to exposure [37].

Regarding mixture effects, studies on dioxins, PCBs, and PBDEs have shown thyroid hormone alterations via reductions in T4, sometimes through dose additivity [28,33,38]. However, this approach may be limited when addressing complex mixtures containing chemicals with dissimilar MoAs. A limitation of the present study is that due to the single dose analyzed, a formal analysis of the nature of the mixture effect is not feasible. 

Notably, compounds C and E primarily exert their effects through the activation of the constitutive androstane receptor (CAR) and/or pregnane X receptor (PXR), whereas prochloraz also functions via the activation of the aryl hydrocarbon receptor (AhR) [33,39,40]. Due to this difference in MIE (CAR/PXR vs. AhR), it is tempting to speculate that UGT induction could deviate from the additivity model. 

In terms of endocrine disruption, there is an increasing body of evidence highlighting the adverse effects of thyroid-disrupting chemicals (TDCs), including azole fungicides, on thyroid function [8,33,41,42]. This correlation follows the characterization of endocrine disruptors that induce changes in TH serum levels and tissues via HPT primary and secondary mechanisms, as suggested by the MoA in our previous study [23], and is supported by EFSA and others [16,27]. This is supported by the Danish Environmental Agency and EFSA’s report on the establishment of cumulative assessment groups (CAGs) to evaluate the effects of pesticides on the thyroid [15,43]. According to the European Commission ED criteria, a compound must have (1) adverse effects, (2) endocrine activity, and (3) a plausible link between the two [44]. However, human relevance must be established for the criteria to be met, or the effects observed are not non-specific secondary consequences of other toxic effects [45]. These categorizations are applied separately to humans and non-target organisms; however, the underlying conceptual framework is the same for both. Existing literature shows azole fungicide effects on model organisms that can be aquatic/non-aquatic non-target organisms [46], but the mixture effects remain a gap in knowledge. Therefore, these results would be of interest in terms of adverse effects not only in the ED context but also in mixture-ED effects with regard to the environment.

Finally, establishing species differences and human relevance is a priority. The thyroid gland is a relatively robust organ that can compensate for mild or moderate disruptions in humans [47]; therefore, some of the effects observed with azole fungicides might be more prominent in other species than in humans. These effects can be confounded by the fundamental differences in thyroid hormone transport between rodents and humans. Rodents primarily rely on albumin and transthyretin for thyroid hormone transport, whereas humans additionally have thyroxine-binding globulin (TBG), resulting in a much shorter serum T4 half-life (approx. 0.5–1 day, versus several days in humans) and greater dependence on hepatic clearance mechanisms (i.e., glucuronidation via UGTs). The larger TBG-bound T4 reserve in humans, on the other hand, can act as a buffer against comparable changes in hepatic enzyme activity [48]. At the receptor level, a previous study by Marx-Stoelting et al. showed that cyproconazole induced PXR/CAR downstream effects in hepatocytes in both murine and human receptors; however, the responses were more pronounced in wild-type mice treated with high doses of cyproconazole [49]. In another review, receptor sensitivity was discussed in the context of azole fungicides, where CAR showed considerable species differences between humans and rodents, whereas AhR or PXR were much less variable. Additionally, some effects were also observed in dogs, showing that the hepatotoxic effects were not rodent-specific, which would echo their importance in supporting a weight-of-evidence assessment for human risk, as cross-species concordance is a key criterion for determining whether observed toxicities may be extrapolated to humans [31].

It is important to note that this study examined a single 28-day exposure duration based on OECD’s TG407 and was consistent with the design of prior single-substance studies. TG407 is intended for hazard identification and endocrine screening but is subject to limitations, including a short time frame, which may miss chronic or delayed-onset conditions that can only be detected in longer-duration tests [50]. This limitation also extends to the mixture interaction observed here.

In conclusion, our findings agree with those of other studies regarding MoA; however, the precise mechanisms underlying these mixture interactions and their implications for human health remain incompletely understood [51]. In developmental toxicology, the total serum T4 level is the primary measurement used to identify TDCs [34]. In this case, the decrease in T4 levels may be related to the azole mixture. However, the interpretation of T4 levels can be challenging, as variations exist in reference ranges for serum T4, as well as the observation that not all xenobiotics known to affect thyroid brain hormone action necessarily reduce T4 concentrations in the blood [52]. A study done by Leemans et al. discussed different mechanisms of thyroid disruption in pesticide mixtures, including TH changes, weight gain/loss, and other KEs as suggested by Noyes et al., but stated that determining the exact mechanism is still difficult as several MoAs could be involved in combined exposures to chemicals [27,41]. This knowledge gap remains important because thyroid hormones play a critical role in organismal development, especially in the nervous system [47,53,54]. However, it is important to distinguish the effects of xenobiotics on adults and in utero, as thyroid hormones play a pivotal role in the developing fetus, and exposure to TDCs may potentially have irreversible health effects.

## 5. Conclusions

Our findings suggest that the mixture effects on the thyroid varied according to the endpoint and biological organization level. UGT induction was observed in all groups but was less pronounced in mixtures than expected, plausibly explaining the differences in the underlying MIEs between prochloraz (also AhR-mediated) and cyproconazole/epoxiconazole (mainly CAR/PXR-mediated). In contrast, TSH elevation was observed only in the mixture groups, and tissue-level morphometric analysis showed that the effects (particularly in the ternary mixture) were not smaller than those of the individual substances. Without formal dose-addition modeling, it cannot be determined whether these mixture responses are additive or less/more than additive from the current data. Notably, the mixture effects in this study were observed at the highest tested dose (NOAELx10), which is substantially higher than the realistic human exposure levels permitted under current regulation. However, direct extrapolation of the present findings to realistic human exposure conditions is limited. The integration of endocrine, hepatic, and structural data elucidates the mechanistic interactions underlying thyroid disruption by azole fungicides and emphasizes the need to evaluate biological layers as well as the human relevance of effects observed in rodent studies when assessing mixture toxicity. A better understanding of if and how TDCs affect thyroid hormone homeostasis may help in predicting the risk of thyroid disease based on patterns of circulating hormone concentrations.

## Figures and Tables

**Table 1:** Morphometry glossary and annotations key (adapted from Kadic et al., 2024 [23]).

Name	Description	Derivation
Epithelial Cell Area	The effective area of epithelial cells	L2–L3
Nuclear Count by Area	Nuclei by thyroid area	Nuclear Count/L1-QuPath
Number of Follicles	Number of annotated follicle lumen in L2	# of L3 annotations
Epithelial cell area by no. of Follicles	Cell area per follicle	(L2–L3)/# of Follicles
Epithelial Cell Area by Follicle Lumen Area	The ratio between the epithelial cell area and the follicle lumen area. Also referred to as the ‘Thyroid activation index’.	(L2–L3)/L3

**Table 2:** Incidence of histological findings in rat thyroid tissue at study termination. Data are shown as the number of animals/numbers examined (percentage). Statistically significant differences (Fisher’s exact test) as compared to the control are indicated by * if *p* ≤ 0.05.

Incidence	Negative Control	MI (C1000 + E900)	MII (C1000 + E900 + P1000)
**Number of animals observed**	6	5	5
**Follicular hypertrophy**	0	5/5 (100%) *	5/5 (100%) *
**Folicular hyperplasia**	0	5/5 (100%) *	5/5 (100%) *
**Follicular dilation**	4/6 (67%)	5/5 (100%)	5/5 (100%)
**No visible lesions**	2/6 (33%)	0	0

**Table 3:** Semi-quantitative scoring of histopathological findings in rat thyroid tissue at study termination. Incidence is indicated by x-marks; group severity scores are the sum of individual animal grades (blue x = slight/1 point, red x = moderate/2 points).

Severity	Negative Control	MI (C1000 + E900)	MII (C1000 + E900 + P1000)
**Number of animals observed**	6	5	5
**Follicular hypertrophy**	0	xxxxx	xxxxx
total follicular hypertrophy score	0	10	10
**Follicular hyperplasia**	0	x xxxx	x xxxx
total follicular hyperplasia score	0	9	9
**Follicular dilation**	xxxx	xxxxx	xxxxx
total follicular dilation score	4	10	10

## Data Availability

The raw data supporting the conclusions of this article will be made available by the authors on request.

## Supplementary Materials

The following supporting information can be downloaded at https://www.mdpi.com/article/10.3390/toxics14090749/s1. Table S1. Dose groups, dietary concentrations and calculated mean daily intakes (based on Schmidt et al. 2016) [21].

## Abbreviations

The following abbreviations are used in this manuscript:

ADME	Absorption, distribution, metabolism, excretion
AhR	Aryl hydrocarbon receptor
AS	Active substance
BCA	Bicinchoninic acid
CAG	Cumulative assessment group
CAR	Constitutive androstane receptor
CAS	Chemical abstract service
CKG	Common kinetic group
CRA	Cumulative risk assessment
CYP	Cytochrome P450
DA	Dose addition
DIO	Deiodinase
EC	Environmental chemicals
EDTA	Ethylenediaminetetraacetic acid
EFSA	European Food Safety Authority
EU	European Union
GLP	Good laboratory practice
HPF	High-power field
HPT	Hypothalamic-pituitary-thyroid
KCl	Potassium Chloride
KE	Key event
MI	Mixture I
MIE	Molecular initiating event
MII	Mixture II
NAM	New approach metholodies
MoA	Mode of action
NC	Negative control
NOAEL	No observed adverse effect level
OECD	Organisation for Economic Co-operation and Development
PBDE	Polybrominated diphenyl ethers
PCB	Polychlorinated biphenyl
PPP	Plant protection products
PXR	Pregnane X receptor
SEM	Standard error of the mean
TBG	Thyroxine-binding globulin
TD	Thyroid disruption
TDC	Thyroid disrupting chemicals
TG	Test guideline
TH	Thyroid hormone
TSH	Thyroid stimulating hormone
UDP	Uridine 5′-diphospho
UDPGA	Uridine Diphosphate Glucuronic Acid
UGT	UDP (Uridine 5′-diphospho)-glucuronosyltransferase
