# Supplementary material for: Mixture Effects Investigation of a Thyroid Hormone System-Specific Interaction with Azole Fungicides
Source: Toxics. 2026 Aug 25;14(9):749. doi: 10.3390/toxics14090749 (PMC13611805; doi:10.3390/toxics14090749)
Supplement: Supplementary file 1 [file toxics-14-00749-s001.zip › toxics-4461426-supplementary.pdf]

1. Animal Experiments

Healthy 6- to 7-week-old male Wistar rats (Crl/Wi) were obtained from Charles River Wiga GmbH (Sulzfeld, Germany). They were acclimatized to conditions of the animal facility for 2 to 3 weeks and continuously checked for health. At the beginning of the feeding study, all animals were 9 weeks old. Animals were caged in groups of five in Makrolon cages (type IV with heightened cover) and received phytoestrogen-free rodent standard diet (R/M-H V155, Ssniff, Soest, Germany) either with or without test substance and filtered tap water ad libitum. Housing and environmental conditions were in accordance with standard operation procedures of the test facility in the German Federal Institute for Risk Assessment (i.e., 20 ± 2 °C, 50–60 % humidity and 12 h dark/light cycle). Environmental enrichment was provided by inclusion of a Rodent Castle (Des. Res. 110698 GLP) and nesting material (paperwool, Art No. 033800, Lilico). As bedding material, approximately 20 g of autoclaved, wooden bedding type S-8/15 (Ssniff) per cage was used. There were five animals in each of the five dose groups per substance and 10 animals per mixture group. Overall, 15 negative control animals were used. Animals were checked daily for clinical signs and mortality. Animals were weighed before the beginning of the experiment, once per week during the experiment and prior to final killing, and blood was sampled from the animals 1 week prior to commencement of treatment and at final killing. When treatment commenced, animals were at an age of 9 weeks, and the group mean bodyweight was 323–350 g with an individual range of 286–385 g. Food consumption was measured daily by weighing the feed for each cage group individually. The principles of the experiment and parameters analyzed were in accordance with OECD TG407, with the exception that the range of organs for subsequent histopathology was limited (see killing and pathology), and the experiment was conducted with male animals only, since males had been shown to be slightly more sensitive in previous studies on which approval of the respective fungicides was based.

2. Study Design and Dose Levels

The individual test substances or mixtures were administered in the diet for 28 days. The original study (Heise et al. 2015) had two parts, where, in Part I, only the effects of five individual test substances were studied. In Part II, mixtures of two or three test substances were applied. The focus of the current work looks into three substances C, E and P, and their respective mixtures, as these chemicals had toxic effects of interest. Mixture I consisted of cyproconazole and epoxiconazole, whereas mixture II contained, in addition, prochloraz. Groups of 10 rats were exposed at three dose levels. Two control groups were included, one for each mixture. Animals belonging to the same group were kept in two neighboring cages. An overview of all treatment groups, intended and actual dietary concentrations, and calculated mean daily intakes is provided in the supplementary table below.

Supplementary Table S1. Dose groups, dietary concentrations and calculated mean daily intakes (based on Schmidt et al. 2016).

Test substances	Nominal concentration [ppm]	Azole content in the diet [ppm] (Mean (±SD))	Deviation from nominal value [%]	Mean daily substance intake [mg/kg bw] (Mean (±SD))
Mixture I - C	1000	895.00 (±123.69)	↓ 10	70.22 (±10.35)
Mixture I - E	900	782.50 (±35.00)[a]	↓ 13	61.39 (±9.04)
Mixture II - C	1000	922.50 (±205.32)[a]	↓ 7	80.63 (±13.56)
Mixture II - E	900	747.50 (91.06)[a]	↓ 17	65.25 (±11.00)
Mixture II - P	1000	717.50 (±38.62)[a]	↓ 28	62.63 (±10.55)

[a] Based on samples taken prior to commencement and at termination of treatment.

3. Sacrifice and pathology

On day 28 of the exposure period, the rats were deeply anesthetized with Sevofluran® (Abbot, Ludwigshafen, Germany) and, after weighing and terminal bleeding, sacrificed by carbon dioxide exposure (95% CO_2 and 5% O_2). Necropsy was immediately performed by a board-certified pathologist who was also responsible for subsequent histopathology. The following organs were taken, weighed and preserved: adrenal glands, heart, kidneys, liver, spleen, prostate gland, and testes. The thyroid gland was trimmed from the trachea and was fixated for further histopathology analysis.

4. Morphometry

On Image Scope, annotations were made in four layers. Layer 1 outlined thyroid tissue at 15x magnification. Layer 2 marked areas with >50% thyroid tissue as HPFs using the "rectangle tool" under 2x magnification. Non-thyroid (i.e., intrathyroidal 'space' located in parathyroid glands) tissue was annotated with the "negative pen tool". Annotated areas were used to compare treatment groups, with positive and negative rectangles contributing to L2 measurement in the glossary (Table 1). Layer 3 identified follicular lumen borders at 20x magnification for follicle counts and lumen area per HPF. Layer 4 used the "ruler tool" at 40x magnification to measure epithelial height. At QuPath, "Brightfield (H&E)" was selected as the image type, and after preprocessing for visual staining quality, thyroid tissue was marked using the 'Brush Tool' at 20x magnification. The preprocessed annotations were analyzed using an automatic cell counter under consistent settings across slides. Figure 2 includes a snapshot of the software and layers used on thyroid samples.
